# Loss of *Kmt2c *in vivo leads to EMT, mitochondrial dysfunction and improved response to lapatinib in breast cancer

**DOI:** 10.1007/s00018-023-04734-7

**Published:** 2023-03-18

**Authors:** Nikiana Simigdala, Anna Chalari, Aimilia D. Sklirou, Evangelia Chavdoula, George Papafotiou, Pelagia Melissa, Aimilia Kafalidou, Nikolaos Paschalidis, Ioannis S. Pateras, Emmanouil Athanasiadis, Dimitris Konstantopoulos, Ioannis P. Trougakos, Apostolos Klinakis

**Affiliations:** 1 Present Address: Biomedical Research Foundation Academy of Athens, Athens, Greece; 2 Department of Cell Biology and Biophysics, Faculty of Biology, National and Kapodistrian University of Athens, Athens, Greece; 3 Department of Cancer Biology and Genetics, The Ohio State University, Columbus, OH USA; 4 The Ohio State University Comprehensive Cancer Center-Arthur G. James Cancer Hospital and Richard J. Solove Research Institute, Columbus, OH USA; 5 2nd Department of Pathology, Medical School, “Attikon” University Hospital, National and Kapodistrian University of Athens, Athens, Greece; 6 Institute for Fundamental Biomedical Research, BSRC ‘Alexander Fleming’, 16672 Vari, Greece

**Keywords:** *KMT2C*, Tumour suppressor, EMT, Breast cancer, Mitochondrial respiration, Lapatinib

## Abstract

Deep sequencing of human tumours has uncovered a previously unappreciated role for epigenetic regulators in tumorigenesis. H3K4 methyltransferase *KMT2C/MLL3* is mutated in several solid malignancies, including more than 10% of breast tumours. To study the tumour suppressor role of KMT2C in breast cancer, we generated mouse models of Erbb2/Neu, Myc or PIK3CA-driven tumorigenesis, in which the *Kmt2c* locus is knocked out specifically in the luminal lineage of mouse mammary glands using the Cre recombinase. *Kmt2c* knock out mice develop tumours earlier, irrespective of the oncogene, assigning a *bona fide* tumour suppressor role for KMT2C in mammary tumorigenesis. Loss of *Kmt2c* induces extensive epigenetic and transcriptional changes, which lead to increased ERK1/2 activity, extracellular matrix re-organization, epithelial-to-mesenchymal transition and mitochondrial dysfunction, the latter associated with increased reactive oxygen species production. Loss of *Kmt2c* renders the Erbb2/Neu-driven tumours more responsive to lapatinib. Publicly available clinical datasets revealed an association of low *Kmt2c* gene expression and better long-term outcome. Collectively, our findings solidify the role of KMT2C as a tumour suppressor in breast cancer and identify dependencies that could be therapeutically amenable.

**Supplementary Information:**

The online version contains supplementary material available at 10.1007/s00018-023-04734-7.

## Introduction

Breast cancer affects millions of women every year [1]. Despite the efficacy of therapies, there is a high degree of variability in response [2, 3]. Several studies [3–7] have performed deep sequencing analysis on breast cancer patient samples to reveal the landscape of somatic driver mutations, which could be associated with tumour aggressiveness and variation in response.

In these studies, among the most frequently mutated genes is the lysine methyltransferase *KMT2C* (also referred to as *MLL3*) [3, 6–8], an epigenetic modifier that methylates the histone 3 tail at lysine 4 (H3K4) at enhancers [9–11] and at specific promoters of genes in a cell-type specific manner [12] as part of the Complex of Proteins Associated with Set1 (COMPASS) [13]. KMT2C is thought to be functionally redundant with KMT2D [11, 12]. KMT2C is often found mutated not only in breast cancer but also in other solid tumours and haematological malignancies [14, 15].

In breast cancer, KMT2C plays a role in the transcriptional control of oestrogen-regulated genes [16–18]. Loss of *KMT2C* correlates with a reduction in proliferation in oestrogen-driven tumour cells but with a promotion of cell growth in oestrogen-depleted media [17]. Importantly, silencing of *KMT2C* in long-term oestrogen deprived cells associates with response to various therapeutic agents such as fulvestrant, AZD9496, ARN1917, GDC927 and RU58668 [17]. In other cancers, KMT2C has been implicated in the therapeutic response to DNA damaging factors or inhibitors of DNA repair proteins [19–21].

Despite the recent interest in KMT2C, its role in breast tumour initiation, progression and response to therapy remains poorly understood. Herein, we assessed the effect of *Kmt2c* loss in mammary tumorigenesis using tissue specific knockout of *KMT2C* locus in various murine models. Our results uncovered a *bona fide* tumour suppressor role for KMT2C in mammary tumorigenesis. Specifically, we show that mammary glands lacking KMT2C activity develop tumours with shorter latency than their wild-type (WT) counterparts. Moreover, inactivation of *Kmt2c* leads to an ERK/MAPK signalling imbalance, dysregulation of mitochondrial respiration and sensitivity to lapatinib.

## Materials and methods

### Mice

*Krt8*^*CreERT2*^*;R26*^*tdTomato*^*;Kmt2c*^*fl/fl*^*;MMTV-Neu*, *Krt8*^*CreERT2*^*;R26*^*tdTomato*^*;Kmt2c*^*fl/fl*^*;MMTV-Myc* and *Krt8*^*CreERT2*^*;R26*^*tdTomato*^*;Kmt2c*^*fl/fl*^*;MMTV-Myc;Pik3ca*^*H1047R*^ mice were used in this study together with control mice that lack the *KMT2C*^*fl/fl*^ component or the Cre driver. The *Kmt2c*^*fl/fl*^ mouse line was generated using CRISPR/cas9 in-house as follows: the W9.5 wild-type 129SV embryonic stem (ES) cells (kind gift from Colin L. Stewart) were cultured in KnockOut DMEM (Gibco, Cat No. 10829018), supplemented with 10% KnockOut serum replacement (Gibco, Cat No.10828028), penicillin/streptomycin (Corning, Cat No. 30-002-CI), nonessential amino acids (Corning, Cat No. 25–025-CI), L-glutamine (Corning, Cat No. 25-005-CI), β-Mercaptoethanol (Applichem, Cat No. A1108) and Leukemia inhibitory factor (LIF) (Santa Cruz, Cat No. sc-4989).

The targeting construct was linearized with Acc65I and phenol/chloroform purified. A mixture of the targeting construct (12.5 μg), the pX330 gRNA 1 (5 μg) and the pX330 gRNA 2 (5 μg) were electroporated into the ES cells that were selected with G418 for 8 days. We added the 2 gRNAs, designed for CRISPR, for increased efficiency.

After selection, a total of 288 resistant clones were picked and the genomic DNA was first screened with PCR genotyping. Thirty-three positive clones were further analyzed by Southern blot, using a 5’ external probe. Genomic DNA was digested with BglII. The targeted allele yielded a band of 2289 bp. Correctly targeted ES clones were injected in C57BL/6 blastocysts. Male chimeras were crossed to C57BL/6 females and offspring was genotyped to assess germline transmission.

The *Kmt2c*^*fl/fl*^ were crossed with mice that carry a *Krt8 CreERT2* driver as well as either the MMTV-Myc or the MMTV-Neu transgenes or the combination of *MMTV-Myc;Pik3ca*^*H1047R*^ (mentioned as MMTV-Myc;ex20 in the manuscript). *CreERT2* was activated by administering tamoxifen (MCE) (a total of 15 mg/25gr distributed over 5 days and administered every other day) to 4 weeks old mice that had reached 18gr of body weight. Tamoxifen (20 mg/ml) was dissolved in corn oil using mild sonication for 30 min with 5 min intervals.

Animals were housed in individually ventilated cages under specific pathogen-free conditions in full compliance with FELASA (Federation of Laboratory Animal Science Associations) recommendations in the Animal House Facility of the Biomedical Research Foundation of the Academy of Athens (BRFAA, Greece). All procedures for the care and treatment of the animals were approved by the Institutional Committee on Ethics of Animal Experiments. The licence for the animal handling protocol for this project is: 1317/13-03-2019. All dissected tumors have comparable size. The size of the tumours was measured using caliper.

### Generation of mouse cell lines

Mice were euthanized, and breast tumour was dissected and washed in ice-cold sterile PBS. Tumour was chopped into very fine pieces using razor blade under sterile conditions and it was incubated with 0.25% Trypsin–EDTA for 15 min at 37 °C. Trypsin was inactivated using DMEM high glycose with 10% FBS. The trypsinized tissue was passed through an 18 G syringe and was let to attach on an FBS-coated plate for 48 h. The plate was split once cells reached confluency. Cells were washed with PBS/5% FBS and stained with anti-CD24 (clone M1/69, Biolegend). CD24 + cells were sorted by flow cytometry (FACSAriaIII, BD) to mark luminal cells and to remove any fibroblasts present.

### In vivo treatments

Lapatinib (MCE HY-50898) was administered by oral gavage daily (200 mg/kg daily) to genetically engineered mouse models (GEMMs). Lapatinib was dissolved in 0.5% carboxymethylcellulose/0.1% Tween-20 using mild sonication for 30 min. Tumour volume was measured twice a week with calliper and calculated as V = axb2/2, “a” being the largest diameter, “b” the smallest. Trametinib (MCE: HY-10999) was dissolved in 40% PEG400, 5% Tween-80, 45% saline and 10% DMSO and was administered orally (1 mg/kg/day).

### Histopathological evaluation

Paraffin-embedded sections were deparaffinized and subjected to Hematoxylin and Eosin (H&E) staining using standard techniques for pathology assessment.

### Immunohistochemistry (IHC) and immunofluorescence (IF)

IHC and IF were performed according to standard procedures. In brief, sections were deparaffinized, followed by heat-induced antigen retrieval with citrate buffer (pH 6) for 20 min using a pressure cooker. In IHC, endogenous peroxidase activity was blocked with hydrogen peroxide for 40 min followed by PBS washes. Sections were permeabilized with PBS/0.3% Triton X-100 and blocked with FBS/BSA/0.3% Triton X-100. Primary antibodies were added for overnight incubation at 4 °C in humidified chambers. The next day, secondary antibodies were added for 2 h at room temperature followed by washes. In IHC, the secondary antibodies (Cell Signaling Technology) were HRP-conjugated and the signal was detected with DAB (Vector Laboratories). Sections were counterstained with Mayer’s hematoxylin and mounted with DPX. In IF, secondary antibodies were purchased from the Jackson laboratory. Sections were counterstained with DAPI (MilliporeSigma) and mounted with mowiol. Primary antibodies: rabbit anti-Ki67 (Abcam ab15580, 1:150), rat anti-KRT8 (Troma I DSHB, 1:100), phospho-ERK1/2 (Cell Signaling Technology #4370, 1:100).

### Immunoblotting

Tumours were lysed in 8 M UREA/50 mM TEAB with protease inhibitors (Calbiochem) using mild sonication on ice followed by homogenization with a 26 G syringe. Lysates were subjected to SDS-PAGE followed by immunoblot analysis. Primary antibodies: phospho-ERK1/2 (CST #4370), total ERK1/2 (CST #9102), phospho- p38 (CST #4511), total p-38 (CST # 8690), GAPDH (CST #5174), H3K4me1 (CST #5326), H3K4me3 (CST #9751), ATP5a (ab14748), NDUFSs3 (ab14711), phospho-c-JUN (sc-822), total c-JUN (CST #9165), phospho-JNK (sc-6254), H-RASs (Millipore), K-RAS (sc-30), KDM6A/UTX (CST #33510), ERα (sc-8002), KRT6α (Biolegend 905702), β-actin (MAB8929, Millipore).

### Real-time PCR

RNA was isolated from snap frozen tumours in TRI reagent (MilliporeSigma) following the manufacturer’s instructions. To assist the RNA isolation, tissue was pulverized with liquid nitrogen using mortar and pestle. cDNA was prepared using PrimeScript RT Reagent Kit (Takara) and quantitative PCR was performed using KAPA SYBR FAST qPCR Master Mix (Kapa Biosystems), in a Roche LightCycler 96. Primer sequences are shown in supplementary Table 1.

### RNA sequencing

RNA was isolated as described above. Initial raw *fastq* files were aligned to the Genome Reference Consortium Mouse Build 38 (*GRCm38*) using the ultrafast universal RNA-seq aligner (*STAR*) (Version 2.7.5b) [22]. Resulted raw counts were generated using the *featureCounts* (Version 2.0.1) [23]. The *fastqc* (Version 0.11.9) and the *multiqc* (Version 1.0) [24] tools were used to access the quality of the alignment and the quantification process. To perform differential expression analysis, DESeq2 [25] was applied. Functional enrichment analysis was performed by using the preranked tool of GSEA [26, 27] and Metascape [28].

### ChIP sequencing

Tumours were dissociated into cells using a collagenase/hyalouronidase mix for 2 h at 37 °C, followed by brief incubation with 0.25% Trypsin–EDTA and then with Dispase as previously described (Prater and Stingl Methods). Cells were then fixed and subjected to chromatin immunoprecipitation as previously described [29] with an additional step of micrococcal nuclease incubation (CST #10011) prior to DNA sonication with metal probe. Antibodies: H3K4me1 (CST #5326), H3K4me3 (CST #9751), H3K27ac (CST #8173).

Raw *fastq* files were aligned to the reference mouse assembly (*mm10*) using the bowtie2 (Version 2.4.1) [30], while the filtering of the uniquely mapping reads was performed using the *sambamba view* (Version 0.6.6) [31]. The bam files of each histone modification were processed with deeptools [32], in order to inspect the degree of correlation between biological replicates, in each condition, genome-wide (3 kb bin counts). The pairs of biological replicates for each condition and histone modification were highly correlated (Pearson Correlation Coefficient > 0.94), thus bam files were down-sampled to the same level of mapped reads, and then merged using samtools [33]. For each IP-INPUT pair, peaks were called by using epic2 [34], an ultra-performant reimplementation of SICER peak caller. To compare the resulting peak sets between biological conditions in an unbiased manner, reads were pre-normalized to the same level of uniquely aligned reads. To scan the mouse genome for wide signal islands, a window of 400 bps with 1 gap was applied. Each read was extended to 200 bps, while the rest of the parameters were set as default. Additional filtering was applied to keep the most significant regions in terms of FDR and log2 Fold Change (FDR < 0.05, and log2 Fold Change > 1) and to exclude all the mm10 blacklisted regions by ENCODE. Peak overlap analysis was performed by using bedTools [35] and intervene python package [36]. Snapshots of bam signal distribution and peaks along particular genomic regions (Atp genes) was visualized using the Gviz package [37].

Peaks were annotated regarding their genomic position relative to genic regions, using ChiPSeeker [38]. To annotate peaks that were exclusively called in each biological condition, bedTools subtract-A was used. H3K4me3 peaks were assigned to gene promoters using bedtools intersect. To define active promoter and enhancer elements, MMTV-Neu H3K27ac peaks were intersected (bedtools) with Ensemble TSSs and FANTOM5 enhancer elements, respectively. H3K27ac, H3K4me3 and H3K4me1 signal was visualized along active promoters (2 kb regions around active TSSs) and active enhancers (2 kb region around enhancer TSSs/eTSSs) as heatmaps and average profiles, using seqMINER [39] and custom R scripts. Pathway enrichment analysis and motif enrichment analysis were performed using ClusterProfiler [40] and i-cisTarget [41], respectively. H3K4me1 exclusive peaks were assigned to candidate cis-regulatory elements (cCREs, The ENCODE Project Consortium, 2020) and were further processed by applying motif enrichment analysis, using HOMER [42].

### Data availability

All the raw sequencing data has been submitted to NCBI SRA portal with the accession ID PRJNA787445.

### Kaplan–Meier plots

Kaplan–Meier survival curves of tumour latency were calculated using the lifelines package (version 0.21.5) in python 3.7. *p* values were calculated using the Mann–Whitney *U* test in scipy package (version 1.2.1) and plots were created using matplotlib (version 3.0.3).

### Clinical analysis

The online tool KM plotter [43] was used to observe statistical significance on RFS in breast cancer patients treated with any therapy. Separate graphs were generated based on the molecular subtype of the patients. The patients were split on median and results with log-rank *p* < 0.05 were assumed significant.

### Mitochondria isolation and measurement of mitochondrial respiration

Mitochondria were isolated as described by [44]. Mitochondrial respiration was determined using a Clark-type O_2_ electrode connected to a computer operated Oxygraph control unit (Hansatech Instruments, Norfolk, U.K.) as previously described [44]. Freshly isolated mitochondria (150 μg of protein) were added to the respiration buffer (120 mM KCl, 5 mM KH_2_PO_4_, 3 mM HEPES, 1 mM EGTA, 1 mM MgCl_2_, 0.2% BSA, pH 7.2) containing 5 mM glutamate/2.5 mM malate. Basal O_2_ consumption was recorded (state 2) and after 2 min 500 μM ADP was added (State 3; indicates rate of ATP production, O_2_ consumption), followed by 6 μΜ oligomycin (State 4; denotes coupling) and 100 nM of the uncoupler (causes maximal respiration) carbonyl cyanide p-trifluoromethoxyphenylhydrazone (FCCP) (State FCCP). In all experiments, the temperature was maintained at 25 °C and the total reaction volume was 300 μl. The respiratory control ratio was calculated as the ratio of State 3 to State 4 (ST3/ST4).

### Measurement of reactive oxygen species (ROS)

For the assessment of ROS production, tissues were incubated with 10 μM CM-H_2_DCFDA (Molecular Probes™/Thermo Fisher Scientific Inc.) dye in PBS for 30 min at 25 °C in the dark. Following dye removal, tissues were incubated for 15 min with PBS, then washed with PBS and lysed in NP-40 lysis buffer. The produced fluorescence was measured in cleared lysates using the Infinite 200 Tecan microtiter-plate photometer (Tecan Trading AG, Switzerland) at excitation and emission wavelengths of 490 and 540 nm, respectively. Obtained fluorescence values were normalized to total protein input.

### Cell culture samples preparation and confocal laser scanning microscopy (CLSM) visualization

To label the mitochondria, cells grown on coverslips were incubated with the MitoTracker™ Green FM (Thermo Fisher Scientific Inc.) probe for 30 min at 37 °C as per manufacturer’s instructions. For visualizing nuclei, cells were counterstained with DAPI (Thermo Fisher Scientific Inc.). Samples were viewed in a Digital Eclipse C1 Nikon (Melville, NY, UAS) CLSM equipped with 20 × 0.50 NA differential interference contrast (DIC), 60 × 1.40 NA DIC Plan Apochromat objectives, using the EZC1 acquisition and analysis software (Nikon).

## Results

### KMT2C is a tumour suppressor in mammary glands of mice and humans

Sequencing data from human primary surgically resected breast tumours [7] identified mutations in the *KMT2C* locus, the majority of which are nonsense [45, 46]. Together with tumours carrying copy number alterations, mostly homozygous deletions, fusions and splice mutations, the cases showing genetic alterations in the *KMT2C* locus comprise 12% of total cases in breast cancer (Supplementary Fig. 1A). The nature of *KMT2C* alterations implies that it likely acts as a tumour suppressor in the mammary epithelium. In agreement with this notion, the disease-specific (DS) and overall survival (OS) of breast cancer patients with KMT2C alterations is significantly shorter (*p* = 0.009278 and 0.0299, respectively, Supplementary Fig. 1B).

**Fig. 1:**
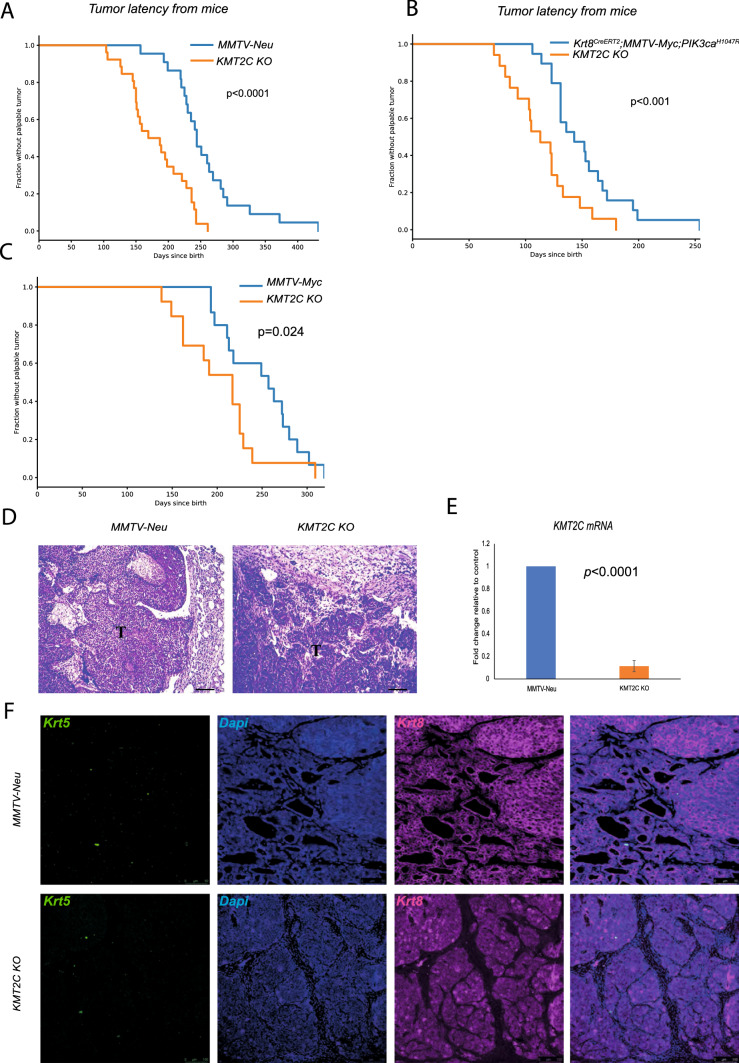
*KMT2C* acts as a tumour suppressor in breast cancer. **A**–**C** Tumour latency from various mouse models with or without *Kmt2c*. **A** *MMTV-Neu* *n* = 22, *Krt8*^*CreERT2*^*;R26*^*tdTomato*^*;Kmt2c*^*fl/fl*^*;MMTV-Neu* (*Kmt2c* KO) *n* = 26, **B **Krt8^*CreERT2*^*;R26*^*tdTomato*^*;MMTV-Myc;PIK3CA*^*H1047R*^ and *Krt8*^*CreERT2*^*;R26*^*tdTomato*^*;Kmt2c*^*fl/fl*^*;MMTV-Myc;PIK3CA*^*H1047R*^* (Kmt2c* KO) *n* = 19 for both, **C** MMTV-Myc, *n* = 15, *KMT2C* KO *n* = 13, **D** H&E staining on MMTV-Neu and *Kmt2c* KO breast tumours, scale bar: 100 μm **E** Representative *Kmt2c* mRNA levels between control and *KMT2C* KO tumours presented as fold change, **F** Immunofluorescence against Krt5, Krt18 followed by Dapi to stain the nuclei on control and KO breast tumours

To investigate its presumptive tumour suppressor role in mice, we employed CRISPR/Cas9 to generate a conditional knockout of the *Kmt2c* locus (Kmt2c^fl^; Supplementary Fig. 2A and B). To delete the *Kmt2c* locus specifically in the luminal lineage of the mammary gland, we employed the Krt8-CreERT2 line [47] (hereafter K8-Cre), which drives the expression of the Tamoxifen-inducible CreERT2 recombinase in the luminal cells. We decided to study the KMT2C role in the context of mouse mammary tumours driven by the well-established breast cancer oncogenic drivers Erbb2/Neu, Myc and PIK3CA^H1047R^. Although mouse models of breast cancer do not faithfully recapitulate the molecular subtypes of the human disease, it has been reported that tumours arising in these models share molecular and histological features with the major human subtypes [48]. We thus generated pairs of cohorts of control and experimental female mice by crossing the Kmt2c^fl^ allele and the K8-Cre transgene into the MMTV-Neu (YD) [49], (hereafter MMTV-Neu) transgenic line, and into bitransgenic mice carrying the MMTV-Myc [50] and the Pik3ca^H1047R^ conditional knock-in mutation (hereafter MMTV-Myc;ex20). In total, we generated three cohorts of experimental mice (*K8-Cre*;*MMTV-Neu*;*Kmt2c*^fl/fl^, *K8-Cre*;*MMTV-Myc*;*Kmt2c*^fl/fl^ and *K8-Cre*;*MMTV-Myc*;a^H1047R^;*Kmt2c*^fl/fl^). As controls, we used littermates lacking either the K8-Cre or the Kmt2c^fl^ alleles depending on the case (for details see Methods section). A reporter transgene (R26^tdTomato^) expressing the fluorescent protein Tomato in a Cre-dependent manner was introduced in all breeding programs employing the Kmt2c^fl^ alleles to monitor recombination [51]. Mice were injected with Tamoxifen five consecutive days starting at the age of four weeks and once they reached a weight of 18 gr. After an 8-week recovery from the hormonal side effects of Tamoxifen, mice were bred for the rest of the monitoring period. Mice lacking KMT2C activity developed palpable tumours invariably earlier, with statistical significance, than the respective controls (Fig. 1A–C). Mice sometimes developed tumours in more than one mammary gland, although no difference in the number of tumours per animal or mammary gland was observed between control and KMT2C KO mice. H&E stainings did not indicate any gross differences between control and experimental mice (Fig. 1D, Supplementary Fig. 2C). Quantitative RT-PCR analysis of tumour samples indicated minimal amounts of *Kmt2c* transcripts, possibly reflecting contaminating stromal cells (Fig. 1E). Cytokeratin profiling of control and *Kmt2c* null tumours showed lack of Keratin 5 (Krt5) and maintenance of Keratin 8 abundance (Krt8) (Fig. 1F). Since the effect of *Kmt2c* loss on tumour latency was more conspicuous (*p* < 0.0001) in the MMTV-Neu mice (Fig. 1A), we focused our mechanistic studies in this model.

### Loss of Kmt2c results in reduction of H3K4me1 and H3K4me3 levels globally

**Fig. 2:**
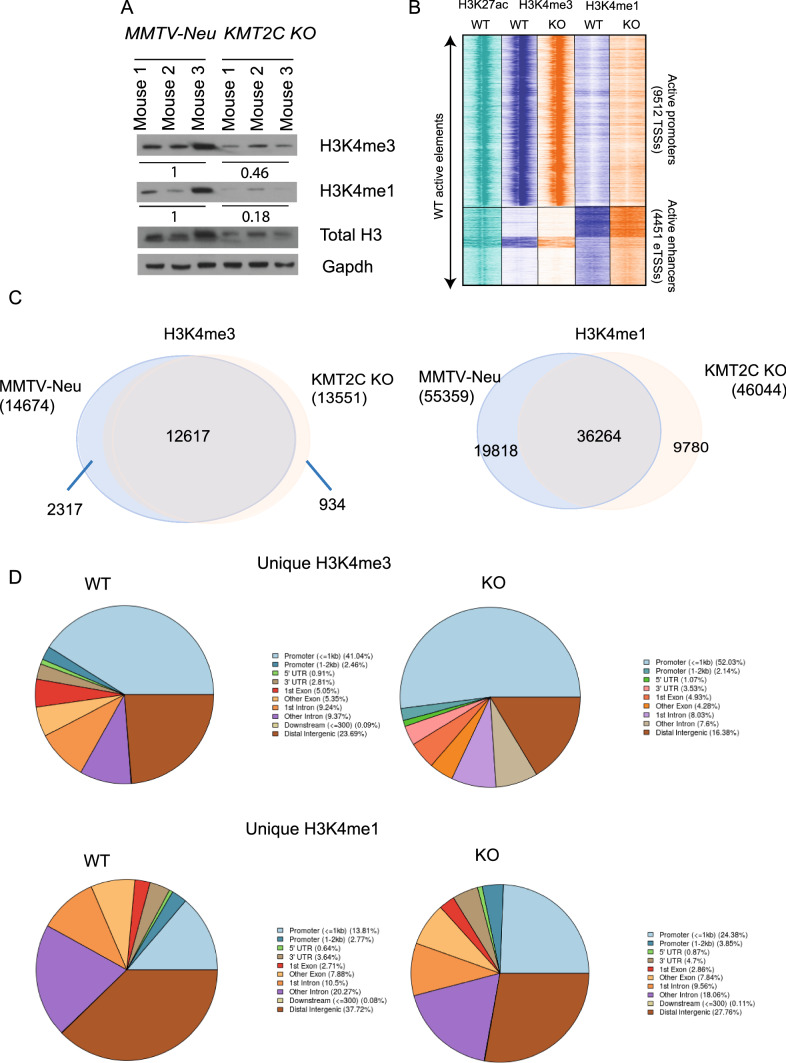
Chromatin distribution of H3K4me3 and H3K4me1 upon *KMT2C* depletion. **A** Immunoblotting analysis of H3K4me3 and H3K4me1 on breast tumour lysates from MMTV-Neu and *Krt8*^*CreERT2*^*;R26*^*tdTomato*^*;Kmt2c*^*fl/fl*^*;MMTV-Neu* (*Kmt2c* KO) mice. Loading normalization was performed by quantifying band intensity in ImageJ. The average of the three bands in control samples was set as 1, and the relative intensity for each antibody in *Kmt2c* KO was found at 0.11, 0.28 and 0.61 for H3K4me1, H3K4me3 and H3, respectively. Normalized signal intensity for H3K4me1 and HeK4me3 in *Kmt2c* KO was calculated at 0.18 (0.11:0.61) and 0.46 (0.28:0.61) for H3K4me1 and H3K4me3, respectively. Individual normalized H3K4me1 values are 0.27, 0.13 and 0.31 for controls, 0.023, 0.08 and 0.02 for knockout mice; for H3K4me3, the values are 1.81, 1.8 and 1.4 for controls and 0.47, 0.96 and 0.67 for knockout mice. Two-tailed *t*-test yields *p* values of 0.028 and 0.006 for H3K4me1 and H3K4me3, respectively. **B** Heatmap of H3K4me3 and H3K4me1 genomic peaks separated into active promoters and enhancers, respectively, by using the pattern of H3K27ac from the MMTV-Neu mouse tumors. **C** Venn diagrams depicting the overlap of H3K4me3 and H3K4me1 between control and *Kmt2c* KO breast tumours, **D** Genomic distribution of the unique H3K4me3 and H3K4me1 peaks for each condition

Although the individual roles of each KMT2 family member are not fully settled, it is generally considered that KMT2C and KMT2D which are closely related to the Drosophila methyltransferase Trr are involved in monomethylation of H3K4 at enhancer sites, while the closely associated trimethylation of H3K4 at transcription start sites is performed by KMT2A and KMT2B, which are homologous to Drosophila Trx [52]. To this end, we carried out ChIP-sequencing on histone 3 lysine 4-mono (H3K4me1) and tri-methylation (H3K4me3)—from MMTV-Neu (hereafter called control) and KMT2C knock out (KO) tumours. Global levels of H3K4me1 and H3K4me3 were significantly reduced in breast tumours from KO mice across all models (*p* = 0.027 and 0.006, respectively), as revealed by immunoblotting analysis (Fig. 2A and Supplementary Fig. 2D). We identified 9,512 active promoters and 4,451 active enhancers using the H3K27 acetylation (H3K27ac), a known mark for active enhancers (Fig. 2B) [53]. In agreement with the immunoblotting analysis, peak calling for the H3K4me3 and H3K4me1 marks showed fewer peaks in the KO compared to control mice. More specifically, we identified a total of 14,674H3K4me3 and 55,359H3K4me1 peaks for the control and 13,551H3K4me3 and 46,044 H3K4me1 peaks for the KO (Fig. 2C). By comparing the overlap of these peaks, we identified 934 unique H3K4me3 and 9,780 unique H3K4me1 in the *Kmt2c* KO (Fig. 2C). The signal around the TSS on the unique *Kmt2c* KO peaks was higher in the KO, while the signal around the TSS for the unique WT peaks was higher in the WT (Supplementary Fig. 3A), a trend also observed for the enhancer transcription start sites (eTSS) (Supplementary Fig. 3B). Furthermore, genomic peak coverage analysis showed that the decrease of H3K4me1 peak regions was corroborated by reduction of H3K4me1 genomic peak coverage in the KO tumours (10% for Neu vs 6% for KO), while the H3K4me1 mark remained essentially stable (1.8% for Neu vs 1.2% for KO).

Genomic distribution of the unique peaks showed that H3K4me3 was enriched at the promoters and reduced at the distal intergenic regions in the KO versus the control. As expected, H3K4me1 peaks were reduced at the distal intergenic regions, possible enhancers, but had an enrichment at the promoters in the KO (Fig. 2D). Genomic distribution of all the peaks followed the same trend but to a lesser degree (Supplementary Fig. 3C). Since, KMT2C and KMT2D are believed to work redundantly, we looked at the levels of *KMT2D* and saw a reduction at the mRNA level in the KO mice. Additionally, mRNA levels of *Wdr5* and *Dpy30*, common core subunits of all COMPASS complexes, were reduced in the KO mice (Supplementary Table 2). Whether this represents an *en bloc* regulation of the genes encoding COMPASS complex components or is merely a by-product of global chromatin remodelling and condensation is a question which warrants further investigation.

### Knock out of Kmt2c leads to disruption of epithelial cell differentiation and an imbalance in ERK/MAPK signalling

**Fig. 3:**
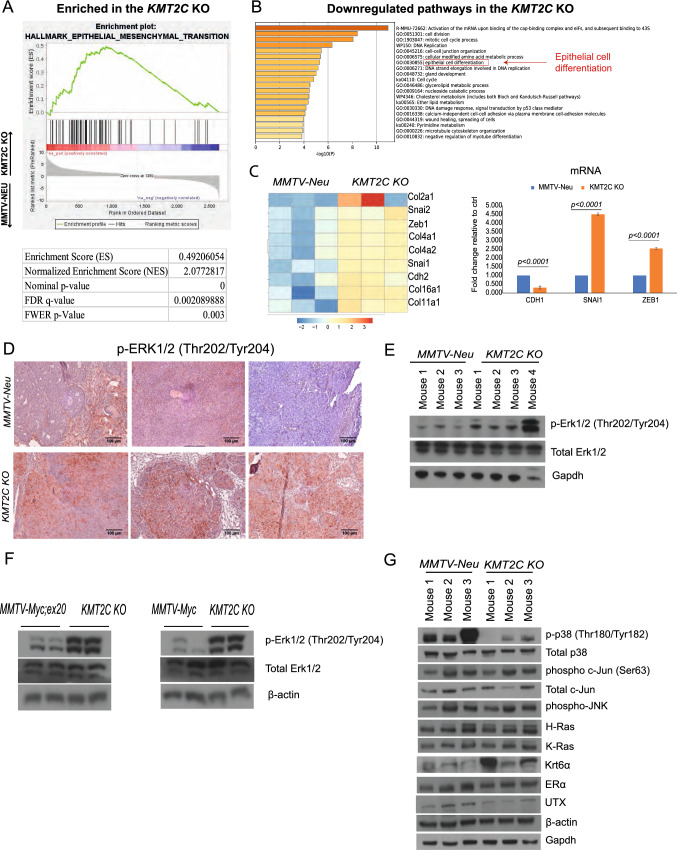
*KMT2C* loss leads to an epithelial-to-mesenchymal (EMT) phenotype and an imbalance in ERK/MAPK signalling. **A** Enrichment of the EMT pathway in the *Kmt2c* KO tumours based on hallmark pathways with GSEA analysis, **B** Downregulated pathways in the *Kmt2c* KO as depicted by Metascape analysis, **C** Heatmap depicting the differential gene expression of genes associated with EMT and one representative qPCR on mRNA levels from *Cdh1*, *Snai1* and *Zeb1* from MMTV-Neu and *Kmt2c* KO breast tumours presented as fold change, **D**–**F** Immunohistochemical and immunoblotting analysis on phospho-ERK1/2 on various mouse models, **G** immunoblotting analysis on various signal transduction proteins. The GAPDH loading control from (**G**) was the same as in Fig. 2A

Since both H3K4me3 and H3K4me1 are histone marks that are associated with active transcription [54], we wanted to assess the potential changes of *Kmt2c* KO in gene expression. Differential gene expression analysis by RNA-sequencing (RNA-seq) revealed 1562 genes upregulated and 1431 genes downregulated significantly (adjusted *p* < 0.05) in the KO as compared to controls (Supplementary Table 2). Pathway analysis using the gene set enrichment analysis (GSEA) and Metascape [28] showed upregulation of genes that promote epithelial to mesenchymal transition (EMT) and downregulation of genes that maintain epithelial differentiation in the KO breast tumours (Fig. 3A and B). We also observed that epithelial cells from KO tumours had more elongated morphology as compared to controls (Supplementary Fig. 4A). The EMT phenotype was corroborated by the higher expression of the EMT-activating transcription factors (*Snai1*, *Snai2* and *Zeb1*) and of the cadherin member CDH2, whose expression is another hallmark of EMT [55, 56]. In addition, various types of collagen proteins (*Col2a1*, *Col4a1*, *Col4a2*, *Col16a1*, *Col11a1*), which disrupt normal epithelial cell differentiation and remodel the extracellular matrix [57, 58], were also upregulated, further promoting EMT (Fig. 3C and Supplementary Fig. 4B). The EMT transcription factors, Snai1 and Zeb1, were also upregulated in the MMTV-Myc and MMTV-Myc;ex20 *Kmt2c* KO mouse models (Supplementary Fig. 4C), further supporting the EMT phenotype in *KMT2C* KO background. Gene annotation and pathway analysis (Supplementary Fig. 5A and B) on the unique identified H3K4me3 peaks from KO tumours corroborated these findings by showing enrichment of Twist1, Twist2 (Supplementary Table 3) and extracellular matrix organization. On the contrary, similar analysis on unique H3K4me3 peaks in the control tumours showed enrichment of epithelial cell differentiation. Furthermore, motif analysis on unique peaks revealed a gain on motifs associated with Nanos1 and C2h2-type proteins. Nanos1 is a transcription factor that depicts inverse correlation with E-cadherin in literature [59]. C2h2-type proteins, include the EMT transcription factors Snai1, Snai2, Zeb1 and Zeb2. Additionally, motif analysis revealed loss of motifs bound by ESE1, an epithelium-specific ETS transcription factor 1, member of the Ets transcription factor family, which negatively regulates ZEBs [60]. Moreover, ESE1, EGR1 and C/Ebpb, which are associated with luminal cell fate commitment are enriched in MMTV-Neu tumors [61–63] (Supplementary Fig. 6).

To investigate the potential changes in signal transduction pathways, we performed staining and immunoblotting analysis on various markers of core signalling cascades and observed increased levels of phospho-ERK1/2 in the KO mice (Fig. 3D–F). Interestingly, both ERK1 and ERK2 have been implicated in promoting EMT in cancer [64, 65].

In support, phospho-p38, another mitogen-activated protein kinase (MAPK), was significantly reduced in the KO tumours as compared to controls (Fig. 3G). Reportedly, p38 has been shown to maintain E-cadherin expression in a HER2 + mouse model [66] and to have opposing activity to ERK signalling in chondrogenesis [67] and neural development [68] along with a suppressive effect on RAS-mediated oncogenesis [69]. Nonetheless, we found no changes in the levels of H-Ras and K-Ras or of phospho-c-Jun and Jnk in our *Kmt2c* KO model (Fig. 3G).

Interestingly, *Kmt2c* KO tumours show higher mRNA and protein levels of the basal cytokeratin Krt6a, which is implicated in EMT [70] (Supplementary Table 2 and Fig. 3G). Moreover, Krt6a expression is associated with basal phenotypes [71], a subtype that has poor clinical outcomes in breast cancer [72, 73]. In line with the Krt6a upregulation, the basal marker Trp63 was also upregulated, while the expression levels of the luminal keratins Krt8/18 were lower in the KO tumours (Supplementary Fig. 5C). Finally, the abundance of oestrogen receptor (ER) was unchanged whereas KDM6A/UTX, a known specific component of the MLL3 COMPASS-like complex, was reduced in the *Kmt2c* KO (Fig. 3G).

### KMT2C KO breast tumours present mitochondrial dysregulation and increased ROS levels

**Fig. 4:**
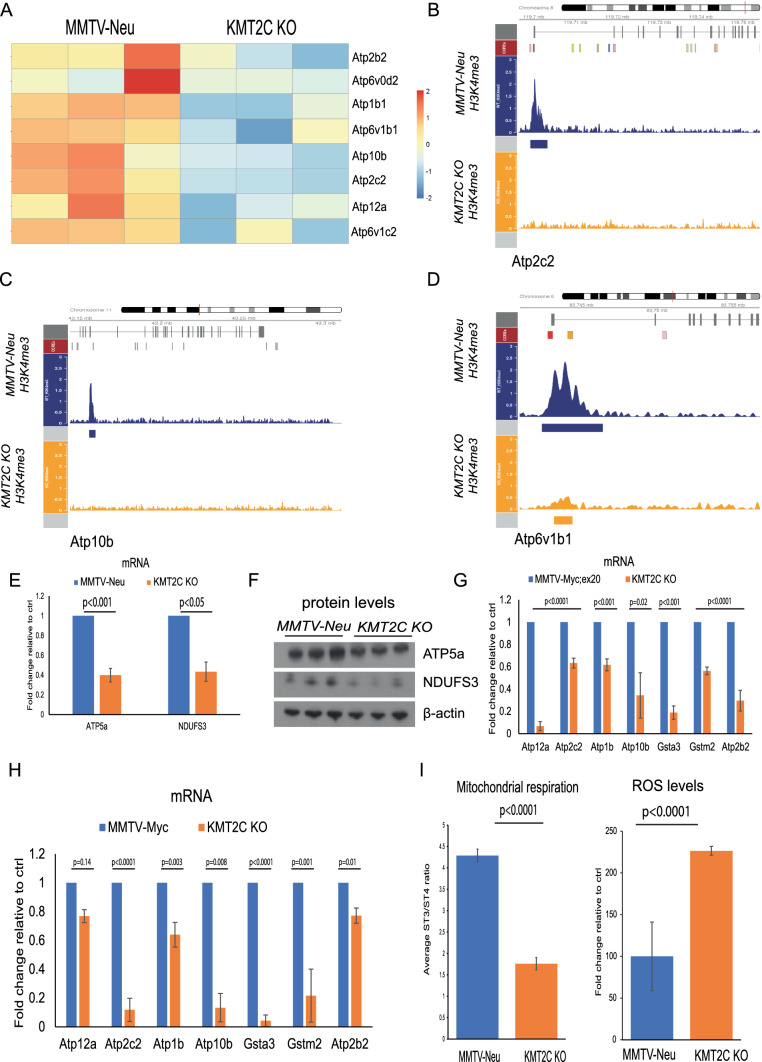
Depletion of *Kmt2c* leads to mitochondrial dysfunction and upregulation of ROS levels. **Α** Differential gene expression on various ATPases also involved in mitochondrial function and oxidative phosphorylation (OXPHOS) from WT and *Krt8*^*CreERT2*^*;R26*^*tdTomato*^*;Kmt2c*^*fl/fl*^*;MMTV-Neu* (*Kmt2c* KO) breast tumours, **B**–**D** H3K4me3 peak pattern on various genes related to the OXPHOS in WT and *KMT2C* KO breast tumours, **E** mRNA levels presented as fold change and **F** protein levels of ATP5a and NDUFS3 from breast tumours, mRNA expression of various OXPHOS-related genes in **G** MMTV-Myc;ex20 and **H** MMTV-Myc control and KO mice, **I** Measurement of mitochondrial respiration and ROS levels from MMTV-Neu and *Kmt2c* KO breast tumours

Our RNA sequencing analysis revealed that expression of ATPase subunits, including members involved in mitochondrial function and OXPHOS, was reduced in the *Kmt2c* KO (Fig. 4A). In agreement with this, our ChIP-seq data showed a remarkable reduction of H3K4me3 at the promoters for many of these genes in the *Kmt2c* KO (Fig. 4B, C and D). Further analysis by qPCR and Western blot of NDUFS3 (complex I) and ATP5a (complex V) showed reduced mRNA (Fig. 4E) and protein (Fig. 4F) levels in MMTV-Neu/KO tumours. Reduction of expression of these subunits was also observed in the MMTV-Myc and MMTV-Myc;ex20 KO mouse models (Fig. 4G and H).

These findings prompted us to measure the mitochondrial respiration rate using freshly isolated mitochondria [44] from breast tumours of control and *Kmt2c* KO mice. Using a Clark-type oxygen electrode, we measured the state 3 (indicates rate of ATP production) and state 4 (indicates coupling) of mitochondrial respiration and calculated the respiratory control ratio (RCR) as the ratio of state 3 to state 4 (ST3:ST4), as previously described [44]. We found that the ST3:ST4 ratio was significantly reduced (*p* < 0.0001) in the *Kmt2c* KO tumours (Fig. 4J-left), which is an indication of mitochondrial dysfunction and damage [74]. Given that mitochondria are major sources of reactive oxygen species (ROS) production as by-product of oxidative phosphorylation [75], we assessed the levels of ROS in our samples and observed a significant increase (*p* < 0.0001) in ROS in the *Kmt2c* KO tumours, further supporting mitochondrial dysfunction, but also a likely deregulation of cellular antioxidant responses (Fig. 4J-right). In support, many genes with antioxidant activity [76], such as *Gstm2*, *Gstt1* and *Gsta3*, were downregulated in the *Kmt2c* KO (Fig. 4G, H sand Supplementary Table 2).

### Loss of Kmt2c is associated with better response to therapy

**Fig. 5:**
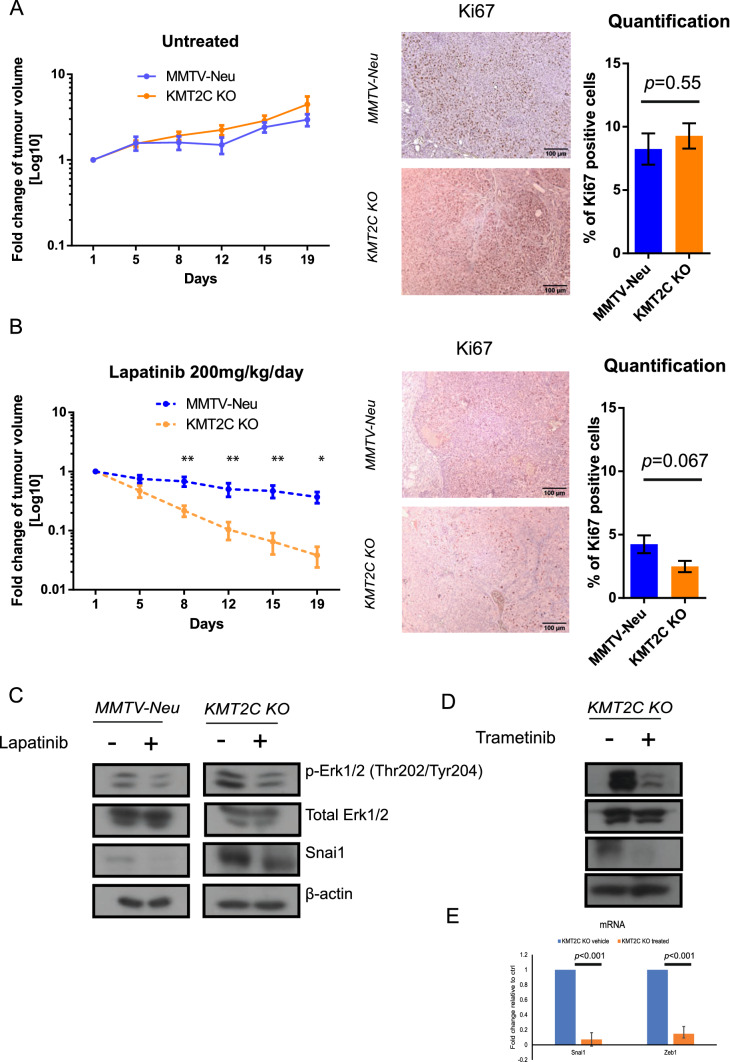
*KMT2C* KO mice are more responsive to lapatinib than their WT counterparts. **A** Tumour growth of untreated mice accompanied with Ki67 immunohistochemistry staining and concomitant quantification (unpaired *t*-test was applied), **B** Tumour growth of lapatinib-treated mice (200 mg/kg/day) along with Ki67 staining and quantification of the Ki67 positive nuclei (unpaired *t*-test was applied). Multiple *t*-test was applied in the lapatinib-treated samples (*p ≤ 0.05, **p ≤ 0.01), The *y* axis of the graphs corresponds to the average values of the fold changes of the tumour volumes for each animal, **C** Immunoblotting analysis of pERK1/2, total ERK and Snai1 on untreated and treated breast tumour lysates. **D** Immunoblotting analysis and (**E**) qPCR on trametinib-treated (1 mg/kg/day) KO mice

To understand whether *Kmt2c* KO tumours grow faster than the controls, we measured tumour growth rate once the tumours were palpable by using a calliper (Fig. 5A). We observed minimal differences in tumour growth rate and Ki67 staining (Fig. 5A-right) between the control and the *Kmt2c* KO tumours (Fig. 5A). Next, we investigated whether *Kmt2c* KO tumours were responsive to lapatinib and found that indeed they were (Fig. 5B). In fact, *KMT2C* KO mice were more sensitive to lapatinib than the MMTV-Neu control ones (*p* = 0.019 at day 19), followed by a marked reduction in Ki67 staining (*p* = 0.067) (Fig. 5B-right). This enhanced oncogene addiction most likely associates with the higher phospho-ERK1/2 levels presented earlier, indicating an increased dependence on the ERK1/2 signalling axis. Because Erbb2/Neu in our mouse model is expressed under the control of the MMTV LTR, and not the endogenous locus, it is unlikely that changes in the expression levels of the Erbb2/Neu oncogene are responsible for this. The downregulation of *Met* and *Erbb3* in the KO tumours (Supplementary Table 2), on the other hand, might contribute to this phenotype. Immunoblotting analysis from mice treated with lapatinib (Fig. 5C) or the MEK inhibitor trametinib (Fig. 5D–E), showed reduction of phospho-ERK1/2 and Snai1 levels, further indicating that EMT is at least in part the result of increased ERK1/2 signalling.

**Fig. 6:**
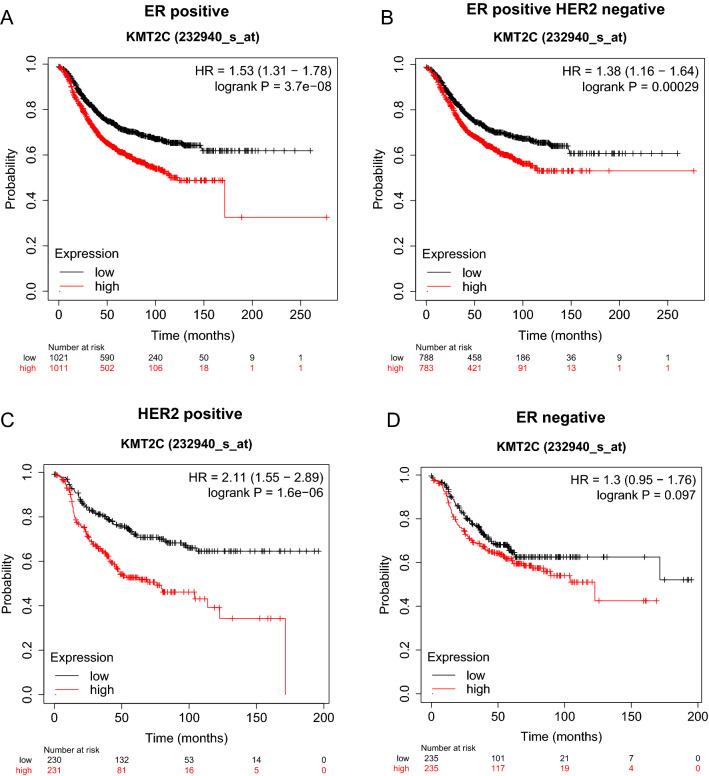
Clinical relevance of *KMT2C* high or low gene expression in various breast cancer subtypes. RFS Data **A** presented from ER positive patients treated with any therapy, **B** ER positive HER2 negative patients treated with any therapy, **C** HER2-positive patients treated with any therapy, **D** ER negative patients treated with any therapy. Red, patients with higher gene expression; black, patients with lower gene expression

To assess the clinical significance of KMT2C, we looked at the relation of *KMT2C* gene expression and the long-term outcome of patients on adjuvant therapy using KM plotter [43]. Low expression of *KMT2C* (probe 232940_s_at) was strongly associated with better RFS on breast cancer patients treated with any therapy (Fig. 6A–D), in this way supporting our mouse data. Combined with literature reports implicating KMT2C in chemotherapy response in hemopoeitic malignancies [77] and solid tumors [20], our findings uncover a broader role for KMT2C in modulating therapeutic response in neoplasia.

## Discussion

It has become apparent that apart from mutations on *PIK3CA* and *TP53* loci, which dominate the mutational landscape of breast cancer samples [6, 7], there is a handful of genes which are recurrently mutated in at least 10% of the cases [6]. One such gene is *KMT2C*, an epigenetic writer of H3K4 methylation. Despite the recent interest in epigenetic modifiers as promising druggable targets in cancer [78], our knowledge on the role of *KMT2C* in breast cancer is still limited.

In this study, we sought to investigate the role of *KMT2C* in breast tumorigenesis and response to therapy using genetically engineered mouse models. In agreement with several lines of evidence from various cancers [79], our study demonstrates that KMT2C acts invariably as a tumour suppressor in breast cancer, since tumours that lack KMT2C became palpable significantly earlier than the WT counterparts. Global levels of H3K4 mono and tri-methylation were reduced in the KO mice. While reduction of H3K4me1 levels is expected, the reduction in H3K4me3 abundance is a paradox, given that this modification is carried out by KMT2A/Menin [12]. This could be explained by the reduction of *Wdr5* and *Dpy30* mRNA levels seen in the *Kmt2c* KO mice, which are known common core subunits of all mammalian COMPASS complexes [80].

While H3K4me3 is associated with active [81] or poised promoters [82, 83], H3K4me1 associates with active or primed enhancers [84] and more recently it was found at the promoters of a subset of silenced genes in a cell-type specific manner [12, 85]. To determine if there is re-distribution of the H3K4 methylation pattern upon KMT2C depletion, we looked at the global genomic distribution of methylation by ChIP-sequencing on H3K4 mono and tri-methylation and observed more peaks at the promoters and fewer peaks at the enhancers for both modifications. The reduction of H3K4me1 at enhancer sites is expected upon KMT2C depletion, but the increase of H3K4me1 at the promoters is a paradox along with the increase in the H3K4me3. Since both H3K4me3 and H3K4me1 are associated with either active or poised states of chromatin, our data cannot exclude the possibility that loss of *KMT2C* leads to perturbation in the methylation states of the chromatin, which then promotes transition to more undifferentiated cellular states and tumour growth. The epigenetic reshaping observed in the *Kmt2c* KO tumours might not be necessarily a direct result of *Kmt2c* loss but could reflect an adaptation process during tumour evolution which is driven by tumour fitness.

To determine if there is dysregulation of transcription upon KMT2C depletion, we conducted gene expression profiling (RNA-seq). Our data revealed that *Kmt2c* KO breast tumours lose their epithelial identity and present an epithelial-to-mesenchymal transition (EMT) phenotype. Although the molecular mechanism is not clear, EMT is associated with downregulation of the transcription factor Grhl2, a known suppressor of EMT in breast cancer [62] and previously found to be enriched in KMT2C binding events [16], and upregulation of EMT-promoting transcription factors such as Snai1, Snai2 and Zeb1 [86–88] as well as EMT target genes such as Cdh2 and various collagens proteins. Pathway and motif analysis on our ChIP-seq data corroborated these findings. These data are reinforced by previous studies conducted in gastric adenocarcinoma [89] and breast [90, 91], where they also observed an association of *Kmt2c* loss with EMT and more aggressive tumour phenotypes. Intriguingly, EMT has been associated with less differentiated stem cells [92]. In concordance with the transition to more undifferentiated states, we found an alteration in the expression of cytokeratins, which serve as markers for the identification of cellular subtypes. Specifically, the mRNA levels of the luminal-specific keratins, *Krt8* and *Krt18*, were reduced in the *Kmt2c* KO, while the levels of the basal-specific keratin, Krt6α were increased. Finally, the levels of Trp63, an important marker of basal identity, stemness and direct transcriptional regulator of basal keratins [93, 94], were increased in the *Kmt2c* KO tumours.

In light of evidence from several studies on the fundamental role of MAPK signalling pathways in cell transformation and cancer [95–97], our results demonstrate a higher abundance of activated ERK1/2 in the KO and a loss of phosphorylated p38. The hyperactivation of ERK in our KO mice could explain the shorter tumour latency, since it is well established that activated ERK is sufficient to initiate cellular transformation [95, 96]. Moreover, ERK1/2 has been shown to induce EMT in various cancer cells acting as the converging hub of upstream activating signals [64, 65, 98, 99]. The finding of phospho-p38 downregulation in our *Kmt2c* KO samples is interesting, since p38 maintains E-cadherin expression [100] and functions as a suppressor of EMT and early dissemination in a HER2 + mouse model [66]. In a recent study, *Kmt2c*^−/−^ mammary outgrowths from organoids showed enrichment in stem-cell-basal markers (CD49^hi^, CD61^+^) and activation of the PI3K pathway [101], an observation we did not see in our mouse tumours.

Furthermore, we discovered that KM2TC regulates the transcription of many subunits related to mitochondrial function and OXPHOS. Upon KMT2C depletion, we observed a decrease in both the H3K4me3 signal and the mRNA expression for many of these genes (Ndufs3, Atp5a, Atp6v1b1). Consistently, we saw a reduction in the mitochondria respiration rate along with increased ROS levels. This finding is reinforced by a previous work on KMT2D, in which a positive regulation of the glutathione detoxification pathway was observed by KMT2D [102]. Additionally, it was recently found that loss of KMT2D leads to deregulation of mitochondrial respiration and enhanced generation of ROS levels [103]. There is a large body of evidence that cancer cells often dysregulate mitochondria and show elevated levels of ROS [104]. While ROS can damage the cell, if they are not properly sequestered in normal physiological conditions, there is evidence that ROS can promote signal transduction in many cancer types [104]. For example, ROS triggered a marked activation of ERK in human glioma cells [105] and mediated degradation of MKP3, a negative regulator of ERK1/2, in ovarian cancer cells [106]. Thus, we cannot exclude the possibility that high ROS levels contribute to the increased phospho-ERK1/2 signal in *Kmt2c* KO tumours.

Finally, our data indicated that *Kmt2c* KO tumours, showed better response to lapatinib. This finding may have therapeutic implications in the clinic, by providing evidence on advantageous treatments to patients carrying *Kmt2c* loss of function mutations. A possible explanation of this finding could be that the genetic ablation of *Kmt2c* does not allow an otherwise expected re-wiring and increased transcription of kinases involved with lapatinib resistance [107–109]. In agreement with this hypothesis, the transcripts of MET and ERBB3 kinases are decreased in the *Kmt2c* KO tumours compared to the controls (Supplementary Table 2). In silico analysis from publicly available clinical samples corroborated these results, showing that *KMT2C* low expression is associated with better long-term outcome.

Taken together, our data demonstrate that KMT2C acts as a strong tumour suppressor in breast cancer. *Kmt2c* KO mice develop breast cancer earlier than their WT counterparts, possibly through the hyperactivation of ERK1/2 and the concomitant suppression of phospho-p38 followed by an activation of the EMT transcriptional program. *Kmt2c* loss leads to downregulation of genes involved in OXPHOS and antioxidant activity, which in turn promotes mitochondrial dysfunction and ROS accumulation, respectively. Importantly, genetic ablation of *KMT2C* sensitised mice to lapatinib treatment. In this sense, it would be interesting to investigate the value of *Kmt2c* status as a biomarker for treatment with anti-HER2 antibodies and/or small molecule inhibitors.

### Supplementary Information

Below is the link to the electronic supplementary material.

Supplementary file1 Supplementary Table 1: Primer sequences used for the qPCR (5’-3’direction) (XLSX 11 KB)

Supplementary file2 Supplementary Table 2: The statistically significant differentially expressed genes as revealed by the RNA-seq data (XLSX 376 KB)

Supplementary file3 Supplementary Table 3: Summary of annotated genes to the unique identified peaks as revealed from the Venn diagram in H3K4me3 and H3K4me1 peaks from controls and Kmt2c KO (XLSX 90 KB)

Supplementary file4 Supplementary Figure 1: Occurrence of KMT2C mutations in humans. (A) Occurrence of KMT2C mutations in 122 out of 996 breast cancer patients taken from the TCGA PanCancer Atlas (cbioportal), (B) Survival data from TCGA PanCancer for breast cancer patients (KMT2C mutated patients=altered group, KMT2C non-mutated patients=unaltered group). The patient cohort is comprised of LumA, LumB, Her2 and basal subtypes. Supplementary Figure 2: Overview of the construct design for the Kmt2cfl/fl mice and H&E stainings from breast tumours. (A) Schematic representation of the construct for the Kmt2cfl/fl mice, (B) Representative southern blot image of the positive clone (c/+) accompanied with a wild type clone (+/+), (C) H&E stainings from breast tumours from MMTV-Myc and Krt8CreERT2;R26tdTomato;MMTV-Myc;Pik3caH1047R along with their Kmt2c KO counterparts, (D) immunoblotting analysis on H3K4me1 and H3K4me3 on MMTV-Myc;ex20, MMTV-Myc control and KO mice. Supplementary Figure 3: Average profiles on TSS and genomic distribution of ChIP-seq. (A) Average profiles at the TSS on unique H3K4me3 and (B) eTSS on unique H3K4me1 identified peaks (C) Genomic distribution of all the peaks from H3K4me3 and H3K4me1 ChIP-seq. Supplementary Figure 4: EMT phenotype in cells derived from MMTV-Neu and Kmt2c KO breast tumours. (A) Confocal images of cells isolated from MMTV-Neu and Krt8CreERT2;R26tdTomato;Kmt2cfl/fl;MMTV-Neu (Kmt2c KO) tumours stained with Dapi and Mitotracker showing the elongated phenotype of the KMT2C KO cells. Based on the cassette that was described in the materials ‘section, the KMT2C KO cells expressed Tomato upon tamoxifen administration, while the control mice not, (B) Snai1 and Snai2 abundances in cells isolated from breast tumours n=2 for each condition and representative qPCR showing mRNA levels of Snai1 in the cells, presented as fold change relative to control. Supplementary Figure 5: Pathway analysis and differential gene expression showing enrichment in extracellular matrix organization and basal markers in the Kmt2c KO tumours (A and B) Pathway analysis on the unique H3K4me3 peaks for each condition using GO and ClusterProfiler, (C) Heatmap showing the differential gene expression of Trp63, Krt8 and Krt18 in Kmt2c KO and control breast tumours as revealed by the RNA-seq data. Supplementary Figure 6: Motif analysis on the unique identified peaks. (A) Motif analysis on unique H3K4me3 peaks and (B) Motif analysis on unique H3K4me1 peaks. (Gained in Kmt2c KO-left, lost in Kmt2c KO-right). (PDF 25701 KB)

## Data Availability

All the raw sequencing data has been submitted to NCBI SRA portal with the accession ID PRJNA787445.
