# Supplementary material for: Loss of Kmt2c in vivo leads to EMT, mitochondrial dysfunction and improved response to lapatinib in breast cancer
Source: Cell Mol Life Sci. 2023 Mar 18;80(4):100. doi: 10.1007/s00018-023-04734-7 (PMC10024673; doi:10.1007/s00018-023-04734-7)
Supplement: Supplementary file 4 — Supplementary file4 Supplementary Figure 1: Occurrence of KMT2C mutations in humans. (A) Occurrence of KMT2C mutations in 122 out of 996 breast cancer patients taken from the TCGA PanCancer Atlas (cbioportal), (B) Survival data from TCGA PanCancer for breast cancer patients (KMT2C mutated patients=altered group, KMT2C non-mutated patients=unaltered group). The patient cohort is comprised of LumA, LumB, Her2 and basal subtypes. Supplementary Figure 2: Overview of the construct design for the Kmt2cfl/fl mice and H&E stainings from breast tumours. (A) Schematic representation of the construct for the Kmt2cfl/fl mice, (B) Representative southern blot image of the positive clone (c/+) accompanied with a wild type clone (+/+), (C) H&E stainings from breast tumours from MMTV-Myc and Krt8CreERT2;R26tdTomato;MMTV-Myc;Pik3caH1047R along with their Kmt2c KO counterparts, (D) immunoblotting analysis on H3K4me1 and H3K4me3 on MMTV-Myc;ex20, MMTV-Myc control and KO mice. Supplementary Figure 3: Average profiles on TSS and genomic distribution of ChIP-seq. (A) Average profiles at the TSS on unique H3K4me3 and (B) eTSS on unique H3K4me1 identified peaks (C) Genomic distribution of all the peaks from H3K4me3 and H3K4me1 ChIP-seq. Supplementary Figure 4: EMT phenotype in cells derived from MMTV-Neu and Kmt2c KO breast tumours. (A) Confocal images of cells isolated from MMTV-Neu and Krt8CreERT2;R26tdTomato;Kmt2cfl/fl;MMTV-Neu (Kmt2c KO) tumours stained with Dapi and Mitotracker showing the elongated phenotype of the KMT2C KO cells. Based on the cassette that was described in the materials ‘section, the KMT2C KO cells expressed Tomato upon tamoxifen administration, while the control mice not, (B) Snai1 and Snai2 abundances in cells isolated from breast tumours n=2 for each condition and representative qPCR showing mRNA levels of Snai1 in the cells, presented as fold change relative to control. Supplementary Figure 5: Pathway analysis and differential gene expression showing enrichment [file 18_2023_4734_MOESM4_ESM.pdf]

Supplementary Figure 1

A

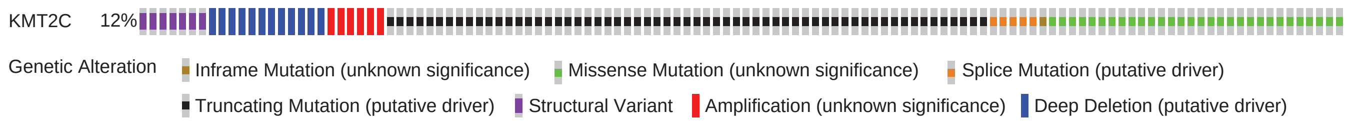

B

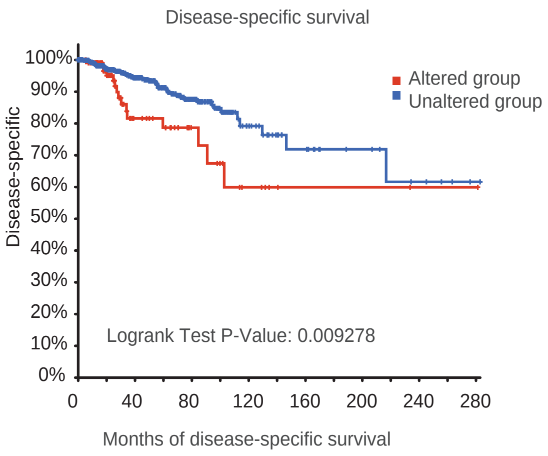

No. of Cases	Total	No. of Events
Altered group	118	15
Unaltered group	859	55

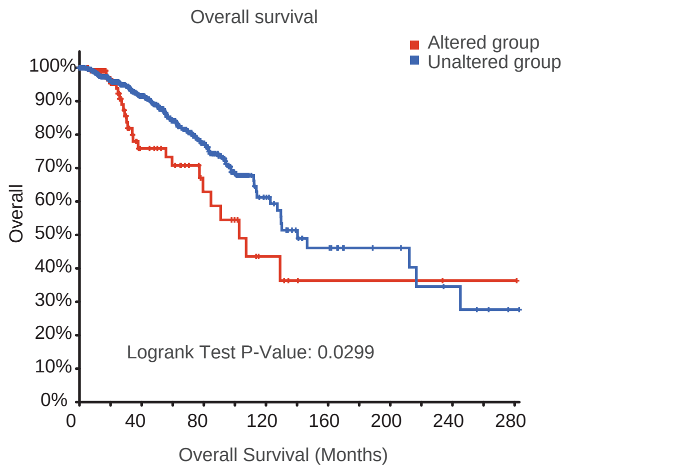

No. of Cases,	Total	No. of Events	Median Months Overall (95% CI)
Altered group	122	24	102.77 (79.46 - NA)
Unaltered group	874	110	140.28 (127.33 - NA)

Supplementary Figure 2

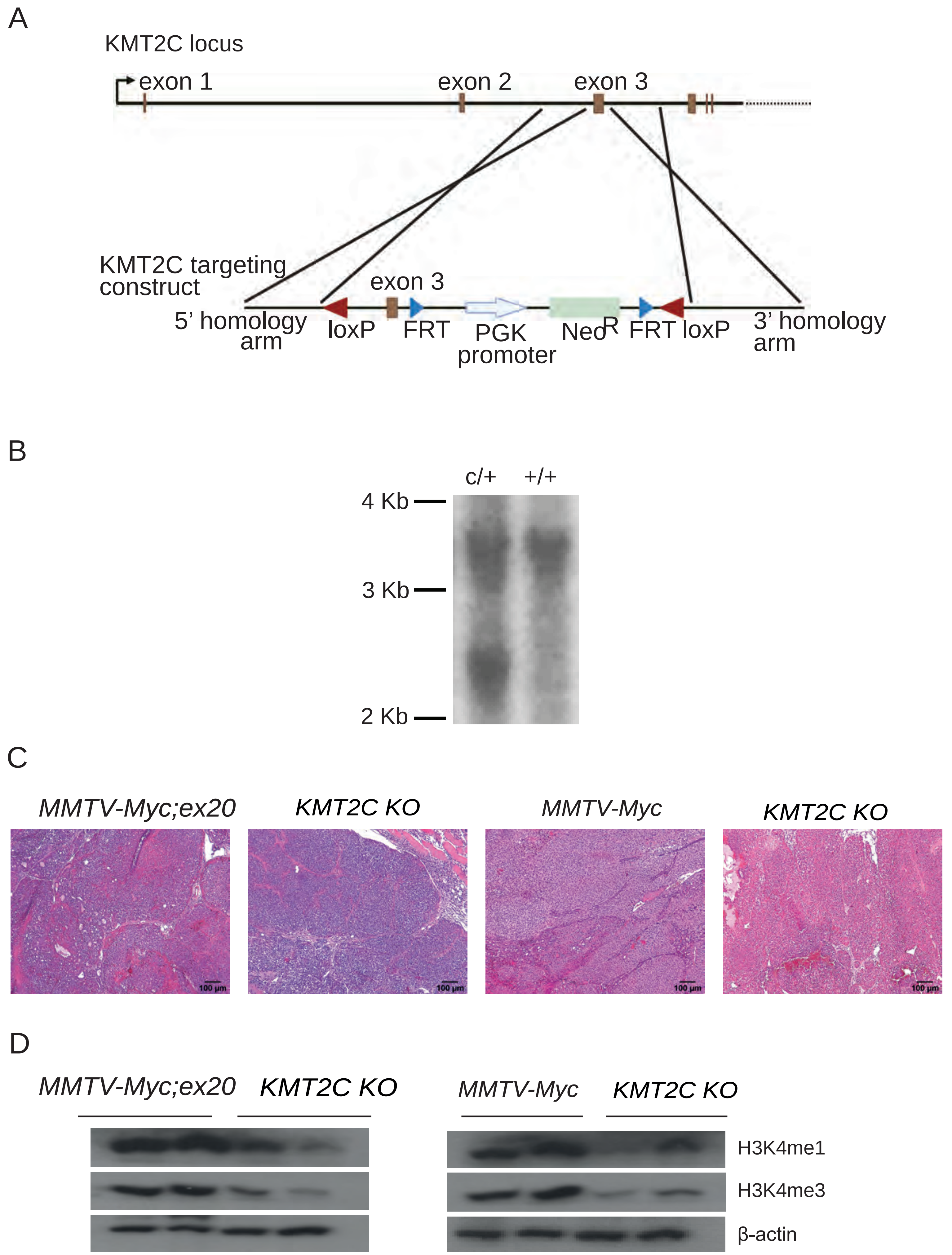

Supplementary Figure 3

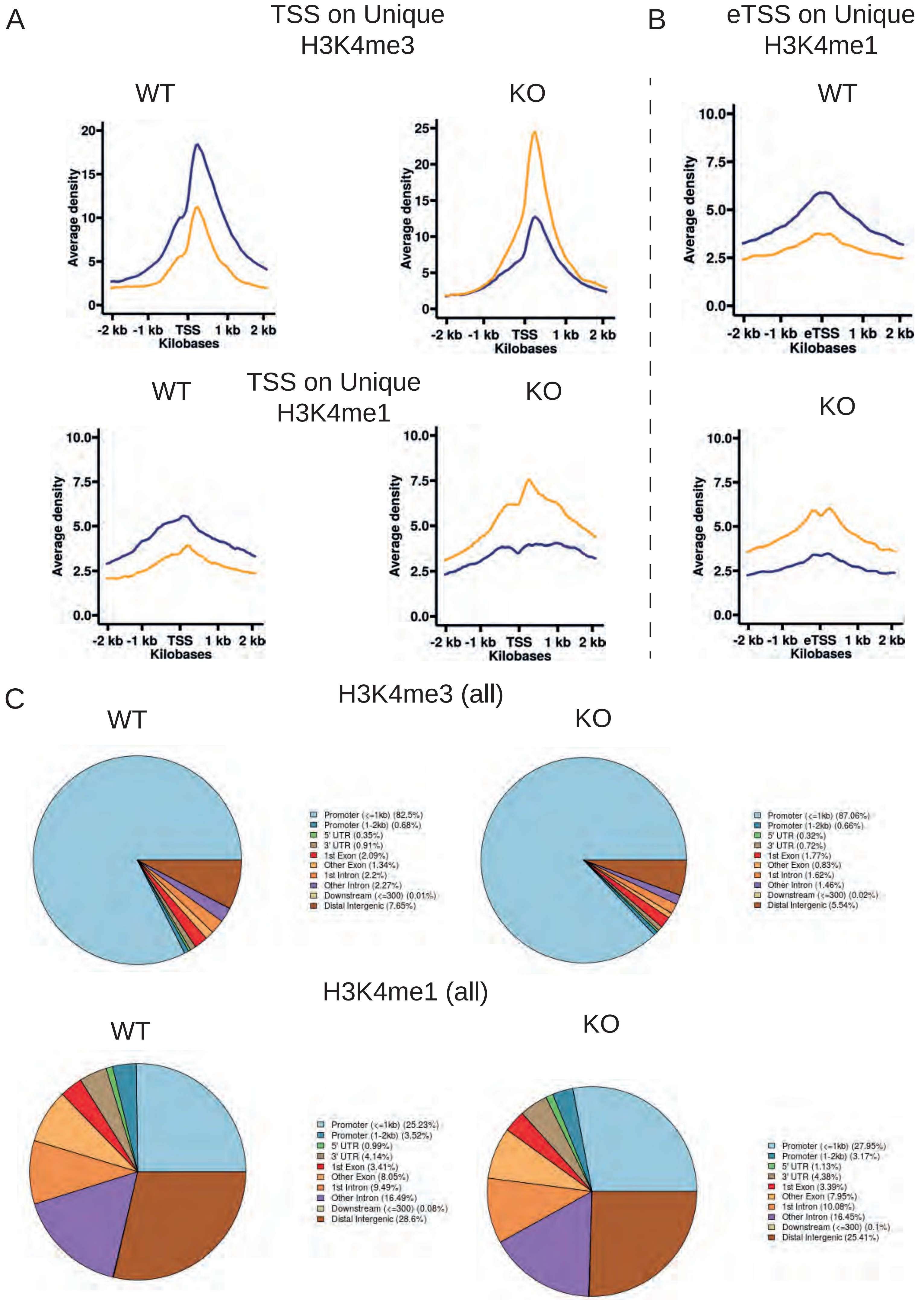

Supplementary Figure 4

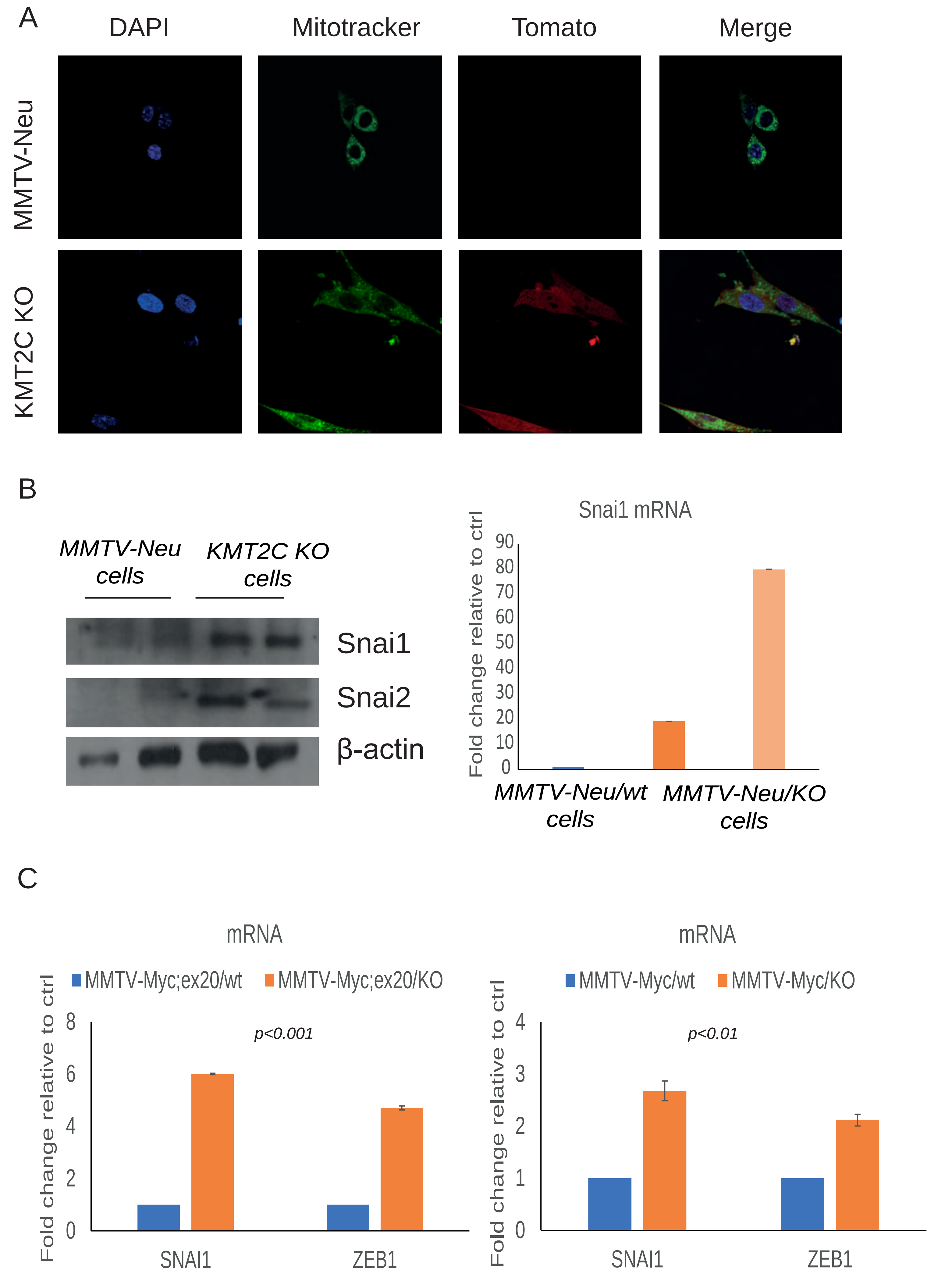

Supplementary Figure 5

A

Pathways enriched in H3K4me3 KMT2C KO

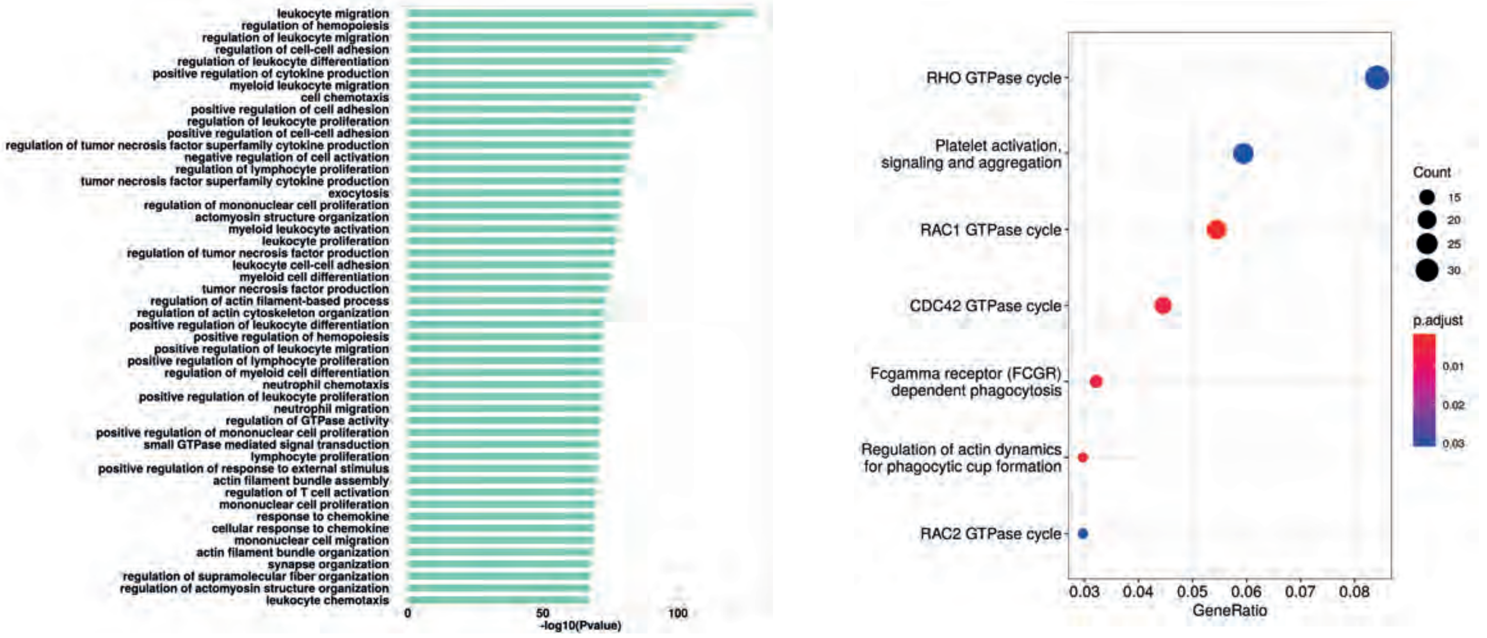

B

Pathways enriched in H3K4me3 MMTV-Neu controls

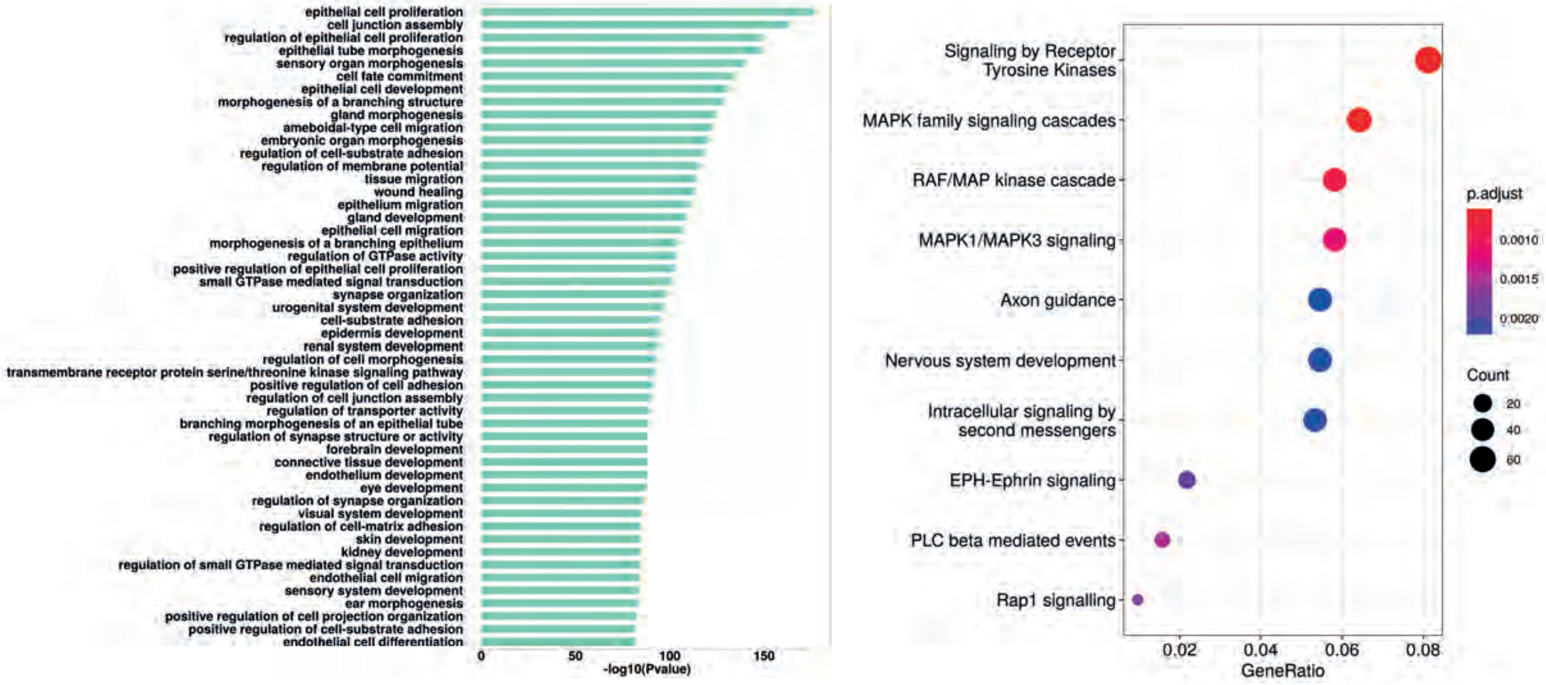

C

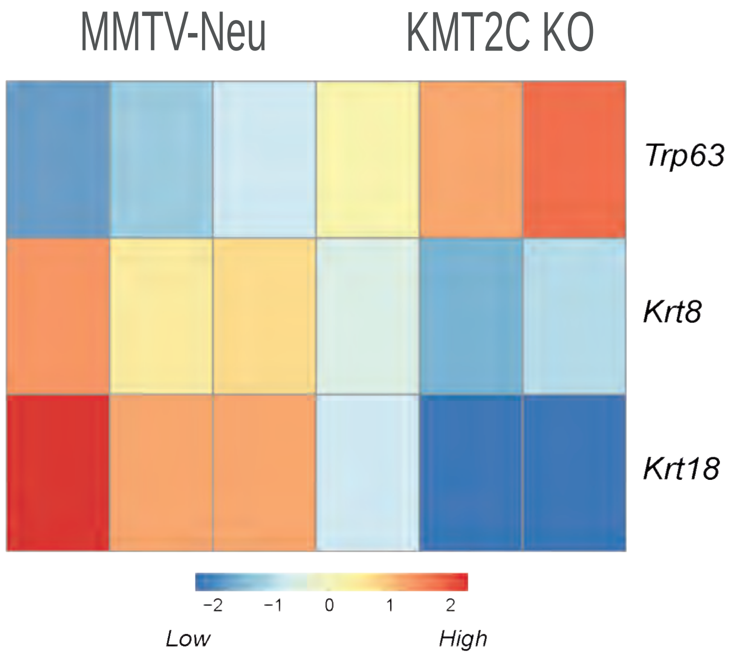

Supplementary Figure 6

Enriched motifs on the unique identified peaks

A

Gained in H3K4me3 *KMT2C* KO

Feature	NES score	Motif
Nanos1	5.22	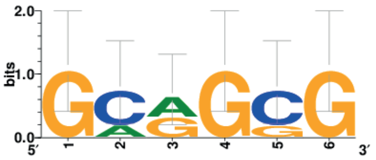
Zf-c2h2	4.36	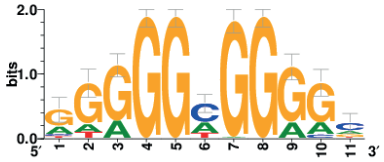

Lost in H3K4me3 *KMT2C* KO

Feature	NES score	Motif
Egr1	3.94	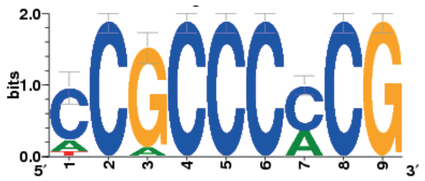
Cebpb	3.65	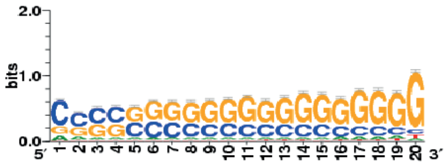

B

Gained in H3K4me1 *KMT2C* KO

Feature	NES score	Motif
Rsc3 (SWI/SNF)	4.52	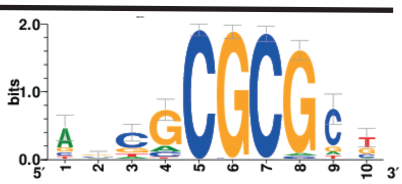
Sp1	4.41	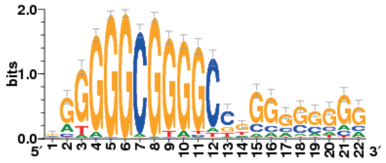

Lost in H3K4me1 *KMT2C* KO

Feature	NES score	Motif
Ese1	3.79	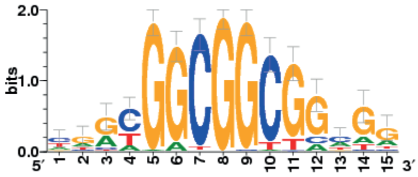
